# An Intelligent Multi-Local Model Bearing Fault Diagnosis Method Using Small Sample Fusion

**DOI:** 10.3390/s23177567

**Published:** 2023-08-31

**Authors:** Xianzhang Zhou, Aohan Li, Guangjie Han

**Affiliations:** 1 Chongqing Academy of Education Science, Chongqing 400015, China; ccqzxz@outlook.com; 2 Graduate School of Informatics and Engineering, The University of Electro-Communications, Tokyo 1828585, Japan; 3 Department of Internet of Things Engineering, Hohai University, Changzhou 213022, China; hanguangjie@hhu.edu.cn

**Keywords:** industrial IoT, bearing fault diagnosis, small sample fusion, transfer learning

## Abstract

It is essential to accurately diagnose bearing faults to avoid property losses or casualties in the industry caused by motor failures. Recently, the methods of fault diagnosis for bearings using deep learning methods have improved the safety of motor operations in a reliable and intelligent way. However, most of the work is mainly suitable for situations where there is sufficient monitoring data of the bearings. In industrial systems, only a small amount of monitoring data can be collected by the bearing sensors due to the harsh monitoring conditions and the short time of the signals of some special motor bearings. To solve the issue above, this paper introduces a transfer learning strategy by focusing on the multi-local model bearing fault based on small sample fusion. The algorithm mainly includes the following steps: (1) constructing a parallel Bi-LSTM sub-network to extract features from bearing vibration and current signals of industrial motor bearings, serially fusing the extracted vibration and current signal features for fault classification, and using them as a source domain fault diagnosis model; (2) measuring the distribution difference between the source domain bearing data and the target bearing data using the maximum mean difference algorithm; (3) based on the distribution differences between the source domain and the target domain, transferring the network parameters of the source domain fault diagnosis model, fine-tuning the network structure of the source domain fault diagnosis model, and obtaining the target domain fault diagnosis model. A performance evaluation reveals that a higher fault diagnosis accuracy under small sample fusion can be maintained by the proposed method compared to other methods. In addition, the early training time of the fault diagnosis model can be reduced, and its generalization ability can be improved to a great extent. Specifically, the fault diagnosis accuracy can be improved to higher than 80% while the training time can be reduced to 15.3% by using the proposed method.

## 1. Introduction

A motor is an important part in machinery as it can provide power, while driving torque in mechanical equipment. Motors are currently widely used in various applications in industrial production, i.e., transportation, aerospace, etc. [1]. If the motor is broken, the operation of the mechanical equipment will be affected directly, which may even cause unpredictable economic losses in the production activities of an entire society [2]. Rolling bearings play an important role because they are critical parts for mechanical equipment in industry. The performance of the control of the motor may be affected and the entire industrial system may be paralysed because of the failure of rolling bearings [3]. However, the mechanical equipment in industrial production is usually installed in harsh environments. The safety of operating the equipment may be affected by faults such as fatigue, pitting, and overload due to the harsh environment [4]. Property damage and casualties will occur once a bearing fault happens [5,6]. Existing works demonstrate that large amounts of system faults are caused by the bearings, both in large and small mechanical systems. Specifically, 40% and 90% for the large and small systems, respectively [7]. Therefore, accurate and real-time motor fault monitoring is very necessary in industrial production [8,9].

In the past few decades, bearing fault diagnosis methods have been extensively studied. Generally speaking, a signal is obtained from a sensor, and sensitively mines and extracts the characteristic signal related to the bearing fault from it. These features are then selected and fused, which can then be used to assess the health of the bearing through a decision-making system. Mechanical fault diagnosis is generally divided into three steps. Signal acquisition is the fist step. Different kinds of sensors are used to collect fault signals. It is important to arrange a limited number of sensors in the most reasonable position for the system [10]. Then, feature extraction followed by feature classification is carried out. The signal processing methods (i.e., wavelet transform, Fourier transform, etc.) are usually combined with traditional fault diagnosis to classify the vibration signal based on a feature classifier after decomposing it in the time and frequency domains [11]. Although many results have been achieved using the traditional fault diagnosis methods, expert knowledge and human experience are necessary for the extraction of artificial features [12,13]. Hence, it is difficult to satisfy the requirements in modern industrial systems [14]. In summary, data-driven methods are more suitable than the traditional spectrum analysis methods based on vibration signals considering the requirements of modern industrial systems [15].

Nowadays, diagnosing bearing faults using deep learning algorithms has become a hot topic. The characteristics of a bearing’s vibrational signals can be learned and modeled by a neural network to diagnose the state of the bearing because of the periodicity of the fault signals of the bearing [16]. Deep-learning-based fault diagnosis methods for bearings have attracted the attention of many scholars in recent years because the data-driven approach avoids the difficulty of complex model building but requires a large amount of historical data [17]. The bearing vibration signal characteristics can be learned and established by a neural network because of the periodicity of the signal. Based on that, the state of the bearing can be diagnosed [16]. Due to the rapid development of deep learning algorithms, as well as their application in image recognition, object detection, and semantics, fault diagnosis methods using deep learning have been intensively studied [9].

Deep learning (DL) technology [18,19] has achieved remarkable achievements in many kinds of rotating bearing status diagnoses including fault diagnosis [20], remaining service life estimation [21], the construction of health indices [22], and so on, in recent years. Diagnosing bearing faults using artificial intelligence methods is well developed because of the rapid development of algorithms based on deep learning. In complex intelligent manufacturing scenarios, it may be difficult to collect a large amount of fault data with high-quality and accurate labeled data for training deep learning models. Particularly, it is always difficult to obtain data of moderate and severe failures in piratical production. Therefore, the lack and imbalance of fault samples will seriously influence the diagnostic accuracy of the trained models [23]. Moreover, it may be difficult to collect massive data samples for some special industrial motors because the duration of the bearing signals of the special motors (i.e., cryogenic pumps, centrifugal pumps, and mine hoist motors) is usually short. In addition, they are used in relatively harsh monitoring conditions. For instance, it costs a lot to troubleshoot subsea production systems, which involves lifting components onto a platform for repair. In this case, the faults cannot be dealt with temporarily if they do not seriously affect the operation of the system. This causes multiple faults to exist at the same time, creating a mixed signal. This makes it difficult to differentiate the status of subsea production systems [24]. Moreover, an overfitting problem may happen during the training of the model when adopting the deep-learning-based bearing fault diagnosis directly, which may reduce the recognition accuracy. It is a key problem and challenge to develop fault diagnosis technologies for motor bearings under small sample conditions while maintaining the accuracy of the recognition.

This paper aims to improve the accuracy of fault diagnosis. Addressing the problem of the low accuracy of bearing fault diagnosis algorithms under small sample conditions. On the basis of a multi-part diagnostic model [25], a migration learning strategy is introduced. Focusing on the similarity and transferability of the multilayer features of industrial motor bearing data, a multi-local model diagnosis algorithm of bearing faults using small sample fusion (MLMF-SS) is further proposed. The contributions of this paper are organized below.

1. To improve the accuracy of fault diagnosis under the condition where only a small amount of monitoring data can be collected due to the relatively short signal duration and the harsh conditions endured by some special industrial motor bearings such as centrifugal and cryogenic pumps, a multi-local model fault diagnosis algorithm of bearing faults using small sample fusion (MLMF-SS) is proposed in this paper. In the proposed algorithm, we consider the similarity and migration of shallow features of industrial motor bearing data by introducing migration learning strategies for solving the low diagnostic accuracy problem with small amounts of data in industry. Specifically, the Bi-LSTM-based multi-local feature fusion model is used as the fault diagnosis model of the source domain. Then, the transfer of a small sample bearing fault diagnosis model based on similarity transfer measurement is optimized. The maximum mean difference algorithm is used to evaluate the data distribution differences between the source domain dataset and the target domain dataset. The network structure and depth of the source domain fault diagnosis model are adjusted based on the evaluation results.
2. The performance of the proposed algorithm is evaluated using an actual experiment platform. Firstly, the effectiveness of optimizing the migration of fault diagnosis models was evaluated. To verify the effectiveness of the proposed fault diagnosis model migration optimization strategy, this paper selects three small-sample processing strategies based on the multi-local feature fusion model for comparison. These are (1) the source domain fault diagnosis model is directly trained using target domain bearing data, termed a conventional strategy (CS); (2) the source domain fault diagnosis model is trained using source and target domain bearing data, termed a mixed strategy (MS); (3) the source domain fault diagnosis model is trained using both the source domain and target domain bearing datasets and all of the network parameters of the source domain fault diagnosis model are fine-tuned, termed the transfer strategy (TS). The experimental results show that the algorithm proposed in this paper achieves higher accuracy compared to the comparison strategy. Then, the proposed MLMF-SS algorithm was evaluated and compared with several advanced algorithms, i.e., long short-term memory (LSTM)-based, transfer component analysis and deep belief network (TCA-DBN)-based, and deep transfer convolutional neural network (TCNN)-based algorithms, under small sample conditions. It is demonstrated that high accuracy and F1 score can be obtained by our proposed algorithm compared with other algorithms.

This paper is structured as detailed below. The related theories, including difference and migration of shallow features of similar bearings and shallow feature migration for bearing faults constrained by small sample constraints, are described in Section 2 and Section 3. Section 4 presents the proposed method. The performance is evaluated in Section 5. Conclusions are provided in Section 6.

## 2. Difference and Migration of Shallow Features of Similar Bearings

### 2.1. Difference of Shallow Features of Similar Bearings

In [26], it is found that relatively basic features can be learned in the shallow network part of the deep learning model. For similar mechanical failure datasets, these shallow features usually have good transferability and strong generality, and can be applied to other similar learning and classification tasks [27]. At the same time as increasing the number of network layers, the deep network in the deep learning model will be more inclined to learn some special features in the mechanical failure dataset to improve the accuracy of classification. Although most industrial motor bearings have certain differences in component systems, introduction time, etc., some industrial motor bearings with similar uses still have many similarities in their overall structure and design parameters. Scholars have found, through in-depth research, that when industrial motor bearings with similar uses have large differences in operating environments and specific models, the shallow features of their condition monitoring data are obviously not highly similar, and show obvious differences. When industrial motor bearings with similar uses are relatively similar in terms of operating environment and specific models, the shallow features extracted from their condition monitoring data are also relatively similar, or even completely consistent. Therefore, this paper deals with the problem of insufficient monitoring data of the bearing condition for diagnosis by transferring the fault diagnosis knowledge of similar industrial motor bearings.

### 2.2. Migration of Shallow Features of Similar Bearings

**Figure 1:**
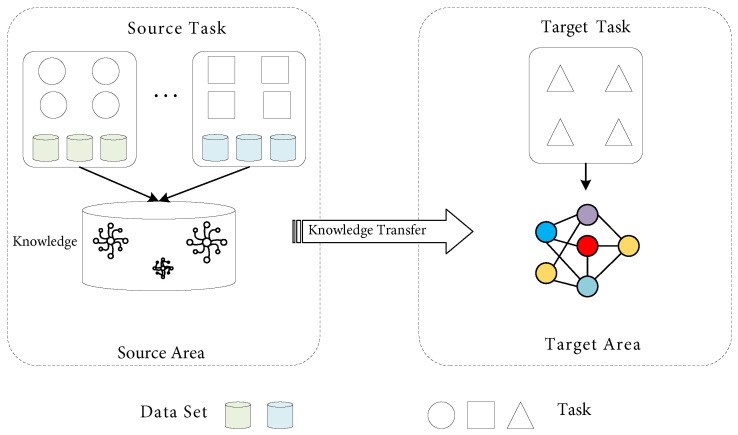
Transfer learning.

Relevant knowledge learned in previous tasks can be applied to other tasks that are not exactly the same, but related, by transfer learning, so as to complete related tasks more efficiently. In transfer learning, the learning goal is generally called a task, and the task mainly includes a label and a prediction function corresponding to the label. Generally, one or more subjects for learning are defined as domains, and each domain has its own feature attribute space and marginal probability distribution. In addition, the object that needs to be migrated is usually called the source domain, which itself has a lot of valuable knowledge, including a large amount of data and detailed annotations. The object that needs to be endowed with knowledge and annotation is termed the target domain. As shown in Figure 1, the process of the transfer will be completed when knowledge is transferred from the source to the target domain. The essence of transfer learning is to optimize the performance of the target domain’s prediction function through the knowledge in the source and target domains.

A novel idea for fault diagnosis of industrial motor bearings with a small sample has been provided by the emergence of transfer learning. However, when the distribution gap of the datasets between the source and target domains exceeds a certain range, directly applying the source domain’s fault diagnosis knowledge to the target domain may reduce the ability of the network to learn for the target domain. However, there must be more or less equal distribution gaps for the dataset between the source and target domains for industrial motor bearings. Therefore, it is difficult to transfer the network structure and shallow features of the source domain directly into the target domain in most cases.

To sum up, the effective measurement of the similarity of the data from the source and target domains becomes the key issue to realize the effective migration of bearing data’s shallow features. Because, only according to the similarity of the data in the source and target domains, can the amount of shallow feature knowledge that can be transferred to the target model be accurately determined. That is, determining which network layers can be migrated, which parameters of the network layers to migrate remain fixed, and which network layer parameters need to be fine-tuned. For this reason, it is necessary to study the migration measurement strategy for fault diagnosis in bearings, so as to maximize the use of common shallow features of the bearing data in the source and target domains.

### 2.3. Migration Selection Based on Sample Dataset Similarity Measure

**Figure 2:**
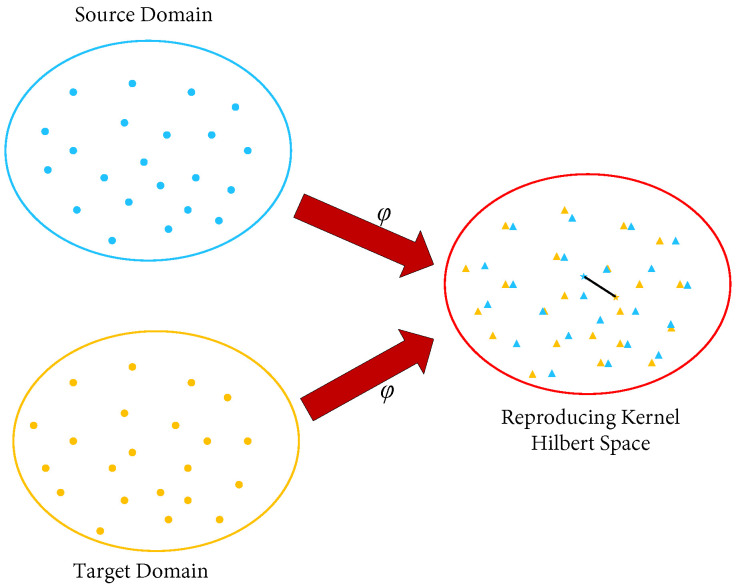
Schematic diagram of the MMD algorithm metric.

In the fault diagnosis process, directly transferring between two domains with unknown similarities or distribution differences will most likely lead to negative transfer of the fault diagnosis model. For this reason, many methods have been used to measure or estimate the distribution differences between different fields. Common measurement methods include Bregman divergence [28], Kullback–Leibler (KL) divergence [29], Jensen–Shannon (JS) divergence [30], and maximum mean discrepancy (MMD) [31]. Among them, Bregman divergence is a sample measure similar to distance estimation. Since it is generally used for solving the function of an objective for methods related to gradient descent, the Bregman divergence will generate a large time cost in the calculation of metrics [32]. KL divergence, also known as KL distance, is a classic sample similarity estimation method. However, this method belongs to the parametric measurement methods, and needs to update the prior probability density continuously during the measurement process. To a certain extent, it increases the amount of calculation and time complexity in the measurement process, which brings great inconvenience to the similarity judgment of the actual engineering dataset. The MMD algorithm is a parameterless distance measurement method, which does not need to pre-estimate the data distribution of the sample before performing the measurement. The method can be viewed as a measure of the distance of the two distributions in a regenerated Hilbert space. That is, the samples are mapped to the reproduced Hilbert space through a special mapping function φ, and then the mean difference of the source and target domains’ data samples on this space is calculated to reflect the difference of the distribution for the two domains, as shown in Figure 2. Compared with Bregman divergence, KL divergence, JS divergence, and other methods, the MMD has the advantages of non-parameterization, a simple and effective calculation, low time complexity, and intuitive judgment, and has become the most widely used metric method in transfer learning. In summary, this paper chooses the MMD algorithm as the measurement method for the similarity migration of shallow features of industrial motor bearings.

## 3. Shallow Feature Migration for Bearing Faults Constrained by Small Sample Constraints

### Similarity Migration Metric of Bearing Shallow Features Based on MMD

At present, the MMD algorithm is always used to measure the difference in the data distribution of the fields. This method maximizes the mean value difference corresponding to the source and target domains using a continuous function, and the maximum mean value difference is the MMD of the distributions for the two domains. The MMD value represents the degree of difference and similarity between the two fields. The larger the MMD is, the greater the difference, as well as the smaller the similarity between the two distributions. The smaller the MMD value, the smaller the difference and the greater the similarity between the two distributions. The main derivation process of the MMD algorithm is as follows:

(1) $$MMD\left[F,p,q\right]=sup(E_{p}\left[f\left(X\right)\right]-E_{q}\left[f\left(Y\right)\right]),$$

where *F* is the projection function space, *f* is the continuous function set of the sample space, *X* and *Y* are the datasets sampled from two different distributions, $E_{p}$ denotes the expected value for dataset *X* after projection, and $E_{q}$ denotes the expected value for dataset *Y* after projection. For relatively complex distributions, The MMD is often estimated empirically based on the datasets that are collected from different distributions. The MMD empirical estimation formula is shown below.

(2) $$MMD\left[F,X,Y\right]=sup\left(\frac{1}{m}\sum_{i=1}^{m}f\left(x_{i}\right)-\frac{1}{n}\sum_{i=1}^{n}f\left(y_{j}\right)\right).$$

The capacities of the *X* dataset and the *Y* dataset are *m* and *n*, respectively. When *F* is a unit sphere on the regenerating kernel Hilbert space, the empirical estimation convergence of the MMD algorithm can be significantly accelerated [33]. The regenerating kernel Hilbert space is mapped by a dot product in the space, as shown in the following equation.

(3) $$f\left(x\right)={\langle f,\varphi (x)\rangle }_{H},$$

Ep[φ(x)] and Eq[φ(y)] can be replaced with $\mu_{p}$ and $\mu_{q}$, respectively, as shown in the formula below.

(4) $$\begin{array}{cc} MMD[F,p,q] & =sup\{E_{p}\left[f\left(X\right)\right]-E_{q}\left[f\left(Y\right)\right]\}\\ & =sup\{E_{p}\left[{\langle \varphi (x),f\rangle }_{H}\right]-E_{q}\left[{\langle \varphi (y),f\rangle }_{H}\right\}\\ & =sup{\langle \mu_{p}-\mu_{q},f\rangle }_{H}\\ & =\;\|\mu_{p}-\mu_{q}{\|}_{H} \end{array}$$

This can be squared and simplified to obtain:

(5) $$\begin{array}{cc} MMD^{2}\left[F,p,q\right] & ={\langle \mu_{p},\mu_{p}\rangle }_{H}+{\langle \mu_{q},\mu_{q}\rangle }_{H}-2{\langle \mu_{p},\mu_{q}\rangle }_{H}\\ & =E_{p}{\langle \varphi \left(x_{i}\right),\varphi \left(x_{j}\right)\rangle }_{H}+E_{q}{\langle \varphi \left(y_{i}\right),\varphi \left(y_{j}\right)\rangle }_{H}-2E_{pq}{\langle \varphi \left(x_{i}\right),\varphi \left(y_{j}\right)\rangle }_{H}. \end{array}$$

The choice of kernel function corresponds to the mapping of high-dimensional space and is of great significance to the subsequent measurement of the distribution difference. The radial basis kernel function has the advantages of a strong mapping ability and wide convergence domain, which is suitable for this task. In addition, the inner product is replaced by $k(x_{i},x_{j})$, as shown in the following formula.

(6) $$k\left(x_{i},x_{j}\right)=e^{\frac{\|x_{i}-x_{j}{\|}^{2}}{2\delta^{2}}}.$$

In summary, the final formula of the MMD can be obtained as:

(7) $$MMD\left[F,X,Y\right]={\left[\frac{1}{m(m-1)}\sum_{i\neq j}^{m}k\left(x_{i},x_{j}\right)+\frac{1}{n(n-1)}\sum_{i\neq j}^{n}k\left(y_{i},y_{j}\right)-\frac{2}{mn}\sum_{i,j=1}^{m,n}k\left(x_{i},y_{j}\right)\right]}^{\frac{1}{2}}.$$

The derivation process of the maximum mean square algorithm can be found in [34,35]. The industrial motor bearing dataset under small sample conditions is applied as the dataset of the target domain while taking a bearing dataset that is similar to the target domain as the dataset of the source domain.

The multi-local feature fusion fault diagnosis model, that is trained in advance using the dataset of the source domain, is used as the source domain fault diagnosis model. Afterwards, the distribution difference of the dataset of the vibrations from the source and target domains, the current source domain dataset and the current target domain dataset is averaged using the MMD final formula in the MMD algorithm and converted into a specific numerical form. This is used as a metric for migration optimization. The similarity-based migration optimization metrics are as follows:

(1) When the average MMD value between the target and source domains is lower than or equal to the set threshold $t_{a}$, the difference between the two domains is minimal, the shallow and deep features between the bearings are highly similar. Therefore, the shallow and the deep feature extraction layers of the fault diagnosis model of the source domain are migrated to generate the fault diagnosis model of the target domain.
(2) When the average MMD value of the target and source domains is higher than the set threshold $t_{a}$ and lower than the set threshold $t_{b}$, it means that the difference between the distributions of the two domains is small while the shallow features between the bearings are highly similar, and the deep features are less similar. Therefore, the shallow feature extraction layers of the fault diagnosis model of the source domain are migrated to generate the fault diagnosis model of the target domain. In addition, a new attention mechanism layer is added in front of the fusion feature layer in the model of the target domain to highlight the contribution of important features to fault diagnosis.
(3) When the average MMD value between the target and the source domains is higher than or equal to the set threshold $t_{b}$, it means that there is a large difference between the two domains while the shallow features and deep features between the bearings have large differences. At this time, it is necessary to re-analyze and process the source domain data, and then perform corresponding processing after determining the applicability of migration optimization. The thresholds $t_{a}$ and $t_{b}$ are set according to expert experience and multiple experimental analysis.

## 4. Bearing Fault Diagnosis Based on Small Sample Fusion

### 4.1. Bi-LSTM-Based Multi-Local Feature Fusion Model Construction

**Figure 3:**
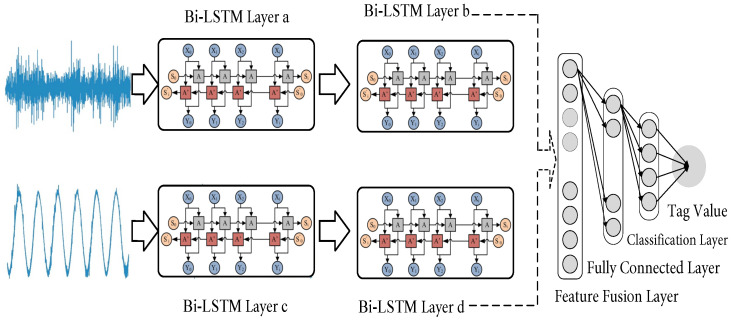
Structure diagram of Bi-LSTM-based multi-local feature fusion model.

This section mainly describes the Bi-LSTM-based multi-local feature fusion model, as shown in Figure 3. The building of the Bi-LSTM-based multi-local feature fusion model is summarized in the steps below.

(1) Selection of the original signal source of the bearing. Considering that there are a lot of uncertainties in fault diagnosis and the complementarity between the current and bearing vibration signals, the current and vibration sensor signals are selected as the original signal source, and used as input information for training the fault diagnosis model in parallel.
(2) Feature adaptive extraction and fusion. Two Bi-LSTM deep learning sub-networks adaptively extract features from the original vibration and current signal sources to deeply mine the deep-level mapping relationship and feature information of the industrial motor bearing vibration sensor signals and current sensor signals in various fault states. After that, the feature fusion layer fuses the vibration signal’s feature information and current signal’s feature information extracted by the Bi-LSTM sub-network. Moreover, in order to prevent overfitting, a dropout operation is added between the feature fusion and fully connected layers. The feature fusion part of the fault diagnosis model considered here does not use more complex feature fusion technology, but chooses direct serial fusion. This is because direct serial fusion has stronger versatility. In addition, no matter whether the feature average summation method or the feature screening fusion method are chosen, it will cause a certain amount of information loss. Direct serial fusion has a lower information loss rate and contains more comprehensive bearing status information. It is obvious to say that fault diagnosis is more suitable under small sample conditions.
(3) Fault identification and classification. The classification and fully connected layers of the fault diagnosis model are mainly used for the recognition and classification stages. In this paper, the features processed by the fusion feature layer are output to the fully connected layer. After performing the conversion of the feature space on the fused features using the fully connected layer, the classification layer is used to identify and classify the bearing status.

### 4.2. Migration Optimization of Bearing Fault Diagnosis Model Based on Similarity Migration Metric

The previous section focused on the Bi-LSTM-based multi-local feature fusion model, which is set as the fault diagnosis model in the source domain of the proposed MLMF-SS algorithm. The model migration optimization process based on similarity migration metrics will be described in detail below.

**Figure 4:**
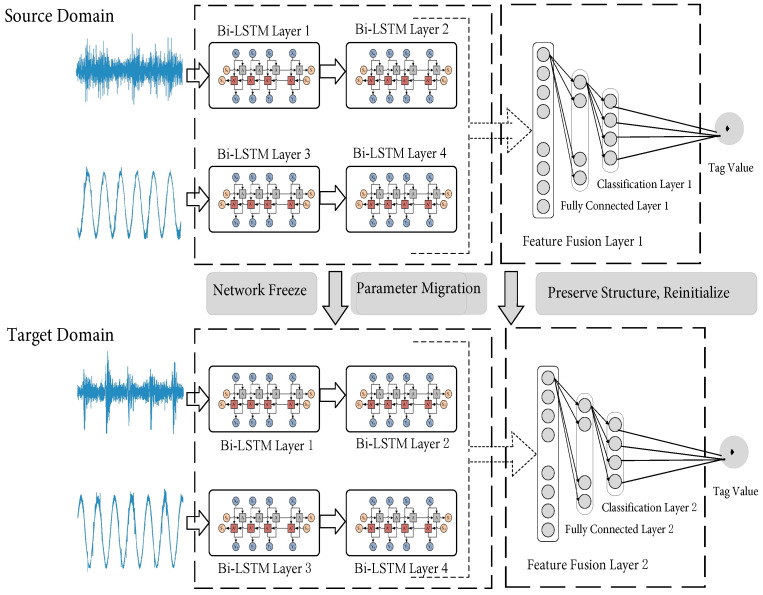
Schematic diagram of migration optimization when the MMD value is lower than or equal to the threshold $t_{a}$.

According to the migration optimization strategy based on the similarity measure, when the average MMD value between the bearing data to be diagnosed (dataset of the target domain) and the selected similar data of the bearing (source domain dataset) is lower than or equal to the threshold $t_{a}$ ($t_{a}>0$), it shows that the distribution of the selected dataset of the bearing in the source domain is very similar to that in the target domain, and the degree of difference is extremely low. There is basically no need to adjust the network structure and network depth of the source domain fault diagnosis model. At this point, the entire network structure and network parameters of the shallow feature extraction part are frozen, i.e., Bi-LSTM layers 1 and 3. Moreover, the deep feature extraction part, i.e., Bi-LSTM layers 2 and 4, in the model of fault diagnosis for the source domain is also frozen. Moreover, it is directly migrated to the bearing fault diagnosis model of the target domain. Meanwhile, the network structure of the feature fusion, fully connected, and classification layers of the model for fault diagnosis in the source domain is retained, and their network parameters are reinitialized. Thus, the bearing fault diagnosis model of the target domain is obtained. The specific migration optimization process is shown in Figure 4 below.

**Figure 5:**
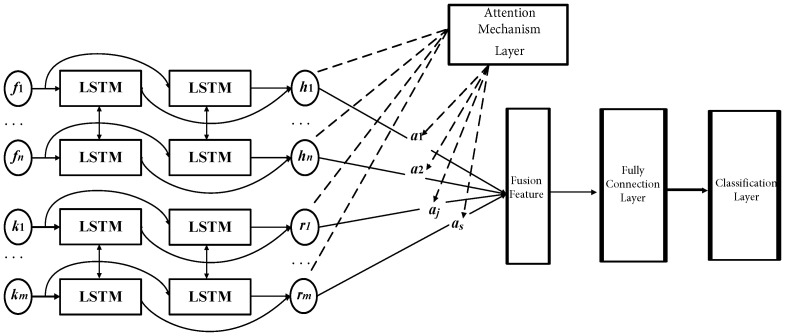
Model network structure after adding attention mechanism.

When the average MMD value between the data of the bearing to be diagnosed (target domain dataset) and the similar data of the bearing (source domain dataset) is higher than the set threshold $t_{a}$ and lower than the set threshold $t_{b}$ ($t_{b}>t_{a}>0$), it indicates that there is a certain difference of distribution for the selected datasets in the source and target domains. Moreover, properly adjusting the network structure and network depth of the fault diagnosis model in the source domain is necessary to generate a fault diagnosis model in the target domain. At this time, the network structure and network parameters of the shallow feature extraction part, i.e., Bi-LSTM layers 1 and 3 in the source domain, are frozen. In addition, they are directly migrated to the bearing fault diagnosis model of the target domain. At the same time, the fully connected and classification layers in the source domain of the bearing fault diagnosis model are retained, and their network parameters are reinitialized. In addition, as shown in Figure 5, a new attention mechanism layer is added in front of the fusion feature layer.

**Figure 6:**
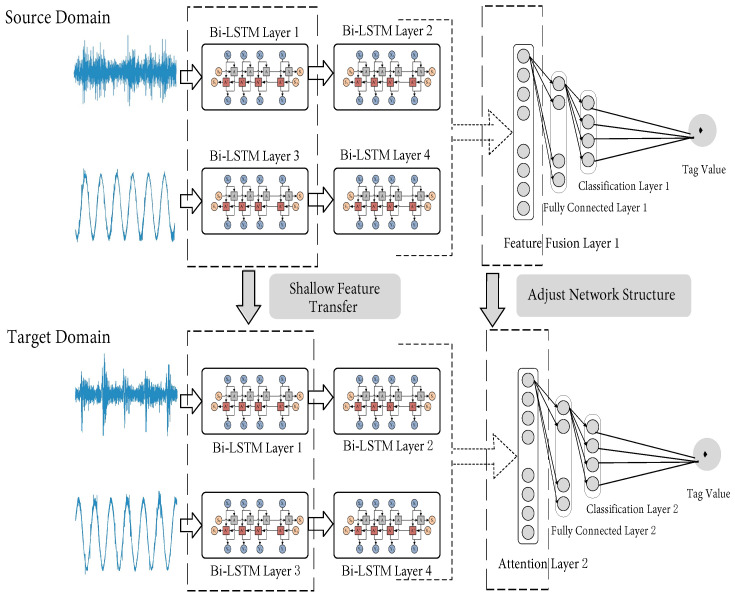
Schematic diagram of migration optimization when the MMD value is higher than the threshold $t_{a}$ and lower than the threshold $t_{b}$.

Although the bearing shallow features of the datasets between the source and target domains have high versatility and similarity, as the degree of difference in the distributions of the source and target domains increases, the similarity of the shallow features between the domains will decrease. This will directly affect the performance of the feature extraction for the model. The attention mechanism highlights the contribution of important features in fault diagnosis by performing adaptive weight matching on the features extracted by the Bi-LSTM. Therefore, the training efficiency and feature extraction ability of the fault diagnosis model are improved. When the average MMD value of the datasets of the bearings in the source and target domains is higher than the set threshold $t_{a}$ and lower than the set threshold $t_{b}$, the migration process is shown in Figure 6.

When the average MMD value between the bearing data that is to be diagnosed (target domain dataset) and the similar bearing data (dataset in the source domain) is higher than or equal to the set threshold $t_{b}$, it means that the similarity between the data in the target domain and that in the source domains is low, but the difference is very large. If the strategy of adjusting the structure of the network while migrating the parameters of the network for the pre-trained model is adopted, it is likely to lead to negative migration. For this condition, it is usually necessary to replace the data samples of the source domain or redesign the fault diagnosis model. However, considering that the source domain bearings selected in this paper are the same or similar to the target domain bearings in terms of type and operating environment, theoretically the degree of difference for the datasets in the source and target domains should not be so obvious. Combined with the similarity measurement strategy using the MMD method proposed in this paper, it is very likely that there are some error samples in the dataset of the source domain. This has a certain impact on the similarity measurement process between the two domains. This may cause the MMD algorithm to misjudge the degree of difference in the distributions between the two domains. To this end, this paper first optimizes the dataset of the bearings in the source domain, and screens the data samples that may have errors in them to rule out the possibility that some samples with error in the dataset of the source domain will affect the similarity measurement results between the source and target domains. The screening process of the source domain data samples is as follows:

(1) After filtering out any data sample w in the bearing dataset in the source domain, calculate the MMD difference $MMD_{w}$ of the two domains using the formula below.
  (8) $$MMD_{w}={\langle \frac{1}{a}\sum_{l=1\;and\;l\neq w}^{a}\varphi \left(A_{i}\right)-\frac{1}{b}\sum_{j=1}^{b}\varphi \left(B_{j}\right)\rangle }_{H},$$
  where *A* and *B* represent the bearing data of the source domain and those of the target domain, respectively. The capacities of the datasets for the two domains are *a* and *b*, respectively.
(2) After that, calculate the average value $MMD_{o}$ of the a $MMD_{w}$, with the calculation formula as follows:

(9) $$MMD_{o}=\frac{\sum_{i=1}^{a}MMD_{i}}{a},$$

(3) Sort all $MMD_{w}$ values in ascending (or descending) order and compare with $MMD_{o}$ values to determine the sample data that needs to be screened out, and generate a new source domain bearing dataset. The judgment formula for dataset sample screening is as follows:

(10) $$\left\{\begin{array}{cc} \lambda =1, & \mathrm{when}\;MMD_{w}<MMD_{o}\\ \lambda =0.5, & \mathrm{when}\;MMD_{w}=MMD_{o}\\ \lambda =0, & \mathrm{when}\;MMD_{w}>MMD_{o} \end{array}\right.$$

where λ is the screening evaluation index.

When λ=1, it means that the sample data are redundant sample data. In order to ensure the data quality of the bearing in the source domain dataset, this sample need to be screened out. After filtering out this sample, the overall distribution difference between the newly generated bearing datasets in the two domains will be reduced.

When λ=0.5, it means that the sample data are normal sample data. It hardly affects the difference of the distribution and similarity of the datasets in the source and target domains before and after sample screening. Selective optimization can be carried out by comprehensively considering the number of samples and the actual situation.

When λ=0, it means that the sample data are valid sample data. To ensure the quality of the dataset of the bearing in the source domain, this sample needs to be kept. If this data sample is filtered out, the degree of similarity between the newly generated bearing dataset in the source domain and the bearing dataset in the target domain will be reduced.

Finally, according to the sample optimization processing results, a new source domain bearing dataset is generated. Then, the MMD value between the bearing dataset in the new source domain and that in the target domain is remeasured to determine the subsequent fault diagnosis process. If the MMD value between the newly generated datasets of the two domains is lower than the set threshold $t_{b}$, it indicates that some wrong data samples are in the bearing dataset of the previous source domain, and these wrong samples or error samples affect the estimation of the MMD value. At this point, it is fine to just follow the fault diagnosis model migration optimization strategy introduced above to complete the follow-up fault diagnosis. If the MMD value between the newly generated dataset in the source domain and that in the target domain is still higher than the set threshold $t_{b}$, it indicates that the degree of difference between the optimized source domain bearing dataset and the target domain bearing dataset is still large. That is, the proposed MLMF-SS algorithm will no longer be applicable. The specific migration optimization process is described in Algorithm 1.
**Algorithm 1** Migration Optimization Process1:Select the bearing dataset in source domain.2:Perform inter-domain similarity transfer metric analysis.3:**if** The number of analyzes is less than *i* **then**4:   **if** MMD value lower than $t_{b}$ **then**5:      Adjust model network structure and transfer parameters.6:   **else**7:      Access channel and used SF are selected using Equation (5).8:   **end if**9:   Optimizing the selected source domain dataset.10: Go to 2.11:**else**12: Go to 1.13:**end if**

## 5. Performance Evaluation

To verify the performance of the method proposed in this paper, this section conducts experiments using the MLMF-SS algorithm based on bearing data and analyzes the experimental results.

### 5.1. Experimental Settings

**Figure 7:**
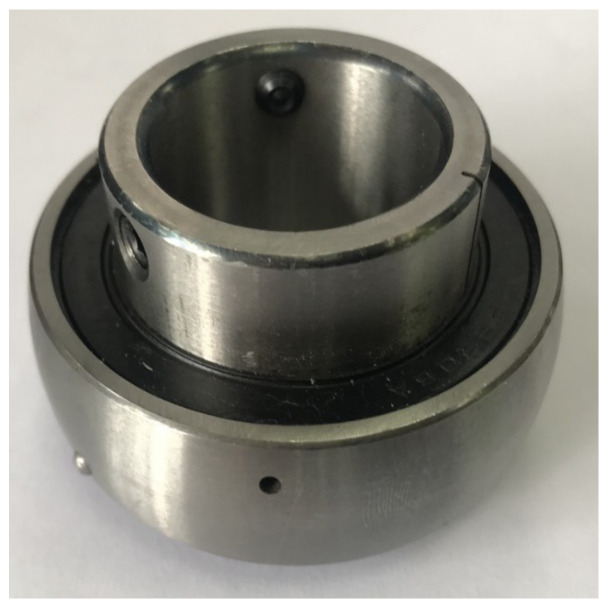
Single-point damage with a diameter of 1 mm.

The experimental platform used in this article is the same as that used in [22], based on the PT700 motor-bearing platform. The experimental platform mainly consists of a three-phase asynchronous AC motor, parallel gearbox, bearing assembly, coupling, frequency converter, oil level gauge, and an electromagnetic powder brake for changing load. In addition, a vibration acceleration sensor was added as the external sensor while a current sensor was added as the internal sensor in the experimental platform. The bearing vibration and current datasets required for the experiment are synchronously collected by VAL-DC29 and VAL-DC25 signal acquisition analyzers. The dataset collected in [25] is set as the bearing dataset of the source domain. Meanwhile, recollect bearing data samples is set as the dataset of the target domain by adjusting the motor speed, motor load, and local single-point damage diameter of the bearing. In order to simulate the failure operation status of similar bearings when there are only small samples, this paper uses the rolling bearing of model PH206, and processes the local single-point damage using EDM technology. The damage locations are located on the outer ring of the bearing and the inner ring of the bearing, and the diameter of the damage is 1 mm, as shown in Figure 7. According to different motor speed conditions, motor load conditions, and local single-point damage diameters, the collected target domain datasets are divided into target domain dataset 1, target domain dataset 2, and target domain dataset 3. Each target domain bearing dataset contains normal status, inner race fault status, and outer race fault status, the same as the operating status contained in the source domain dataset. Each target domain dataset contains 300 samples, and each running state consists of 100 samples. To ensure that the periodic characteristics, as well as the fault information of one bearing revolution, are contained in the collected data, each sample contains the sampling points of one rotation for the tested bearing. It can be calculating that the number of samples for one revolution is around 512 points by using the relevant highest analysis frequency and transmission ratio formula. That is, it is set that each sample contains 512 consecutive sampling points. At the same time, the entire target domain dataset is divided into a training set and a test set at a ratio of 1:1. That is, both the training and test sets in the dataset of the target domain consist of 150 samples. The specific bearing source domain dataset and target domain dataset information is shown in Table 1.

The fault diagnosis model of the source domain mainly includes a parallel Bi-LSTM overlay layer, a feature fusion layer, and fully connected and classification layers. Wherein, each Bi-LSTM superposition layer includes two Bi-LSTM layers (Bi-LSTM layer a and Bi-LSTM layer b). First, raw bearing vibration and current sources are fed directly into a parallel Bi-LSTM overlay. Then, the Bi-LSTM deep learning sub-network deeply mines the deep-level mapping relationship and feature information of the vibration sensor and current sensor signals of the bearings in each fault state. Afterwards, the feature fusion layer directly fuses the bearing’s deep features extracted by the parallel network in series. Finally, the vector output of the feature fusion layer is input to the fully connected layer. These nonlinear feature vectors are weighted, and the predicted category of the target state will be realized as the output of the classification layer. The network structure parameters of the fault diagnosis model of the source domain are given in Table 2.

The experiments are implemented by programming under the framework of TensorFlow. In terms of the learning rate, the dynamic adjustment strategy is still selected, which is realized through the Adam adaptive optimization algorithm. Due to the migration optimization strategy proposed in the proposed method, part of the structure and network parameters of the source domain network will be migrated to the target domain to be fixed. Therefore, there is a relatively small change in the network parameters for the model of the target domain in theory. So the initial learning rate set in this section is 0.0005. In addition, the loss function is set as the cross-entropy, which effectively solves the problem of slow updates of the weight of the square difference loss function, which can be expressed as follows.

(11) $$L=\sum -\left[c_{i}In\left(c_{i}^{*}\right)+\left(1-c_{i}\right)In\left(1-c_{i}^{*}\right)\right],$$

where *L* denotes the loss function, $c_{i}$ is the actual operating state for the bearing, and $c_{i}^{*}$ is the bearing state predicted by the fault diagnosis model. In terms of performance evaluation indicators, the F1 score indicator as well as the accuracy of diagnosing the fault are measured.

### 5.2. Effectiveness Evaluation of Fault Diagnosis Model Migration Optimization

In order to compare the degree of difference more intuitively between the bearing data in the source and those in each target domain, we convert the degree of difference between the domains into the form of the MMD value. The purpose of using datasets containing different speeds, loads, and damage diameters is to evaluate the effectiveness of the maximum mean deviation algorithm in our proposed method. Specifically, the MMD values of the source domain and the target domain bearing datasets 1, 2, and 3 that we used were 0.110, 0.382, and 0.857, respectively. Combined with Table 1, it can be found that as the difference of the experimental conditions for the source and target domains increases, the MMD value also increases. That is, the similarity between the two datasets becomes lower and lower. Compared with the experimental conditions of the source domain, when using dataset 1 for the target domain, the motor running speed of the experimental platform is only slightly increased, and the rest of the experimental conditions are almost identical. When using dataset 2 for the target domain, in addition to slightly increasing the motor running speed of the experimental platform, the load of the motor was also increased. When using dataset 3 for the target domain, not only the motor running speed and load capacity of the experimental platform were improved, but also the local damage diameter of the bearing was changed. Therefore, the difference between the distribution in dataset 1 in the target domain and that in the source domain is the smallest. Meanwhile, the difference between the distribution in dataset 2 in the target domain and the dataset in the source domain is small. Moreover, the difference in the distribution in dataset 3 in the target domain and that in the source domain is the most obvious. In addition, based on relevant experience, the migration optimization thresholds $t_{a}$ and $t_{b}$ in this section are set to 0.15 and 0.65, respectively. To evaluate the rationality of the model migration optimization strategy for fault diagnosis, the MLMF-SS algorithm is subdivided into three parts. The first part, dealing with the situation where the average MMD value between domains is lower than or equal to the threshold $t_{a}$, is denoted as transfer optimization algorithm A (TOA-A). The second part, the situation where the average MMD value between the domains is higher than the threshold $t_{a}$ and lower than the threshold $t_{b}$, is denoted as transfer optimization algorithm B (TOA-B). The third part, the case where the average MMD value between the domains is higher than or equal to the threshold $t_{b}$, is denoted as transfer optimization algorithm C (TOA-C).

**Figure 8:**
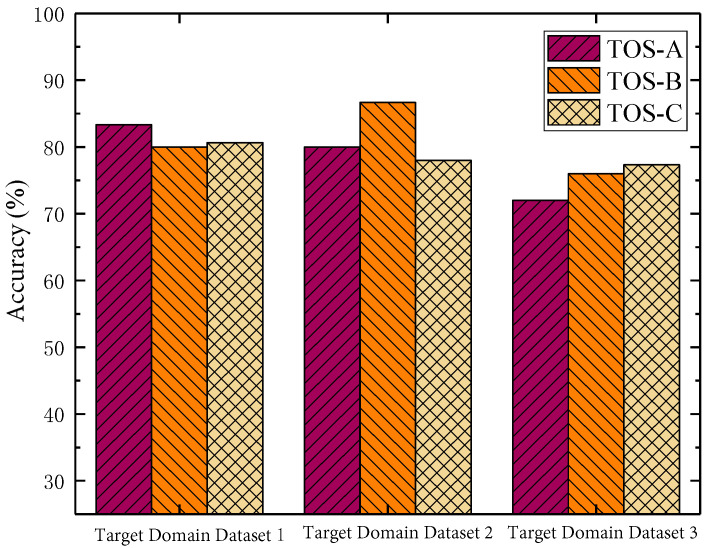
Fault diagnosis accuracy of different algorithms.

Figure 8 shows the accuracy of the optimization algorithms in fault diagnosis. The TOA-A algorithm, the TOA-B algorithm, and the TOA-C algorithm have achieved the best results in their respective advantageous processing ranges. Among them, the accuracy rate of the TOA-A algorithm is as high as 83.33% when using dataset 1 in the target domain for experiments. The accuracy rate of the TOA-B algorithm is as high as 86.67% when using dataset 2 in the target domain for experiments, The accuracy of the TOA-C algorithm is as high as 77.34% when using dataset 3 in the target domain for experiments. This shows that the MLMF-SS algorithm proposed in this section is reasonable. At the same time, it also proves that there is a certain correlation between the accuracy that can be achieved by the fault diagnosis algorithm and the maximum mean difference between fields.

It is worth noting that the TOA-C algorithm still screens and optimizes the dataset of the bearing in the source domain when the difference in the distributions of the datasets between the source and the target domains is small. At this time, the screening and optimization of the dataset may not improve the quality of the dataset. On the contrary, some data samples may be deleted by mistake, which will damage the bearing data including the timing characteristics, and finally affect the accuracy of fault diagnosis.

In the performance evaluation, the model of the multi-local feature fusion fault diagnosis is set as the model of fault diagnosis in the source domain. The strategy for directly using the data in the target domain to train the fault diagnosis model in the source domain and perform fault diagnosis with a small number of samples is called the traditional strategy (conventional strategy, CS). The strategy of using the datasets in the source and target domains to jointly train the fault diagnosis model for the source domain and perform the fault diagnosis using a small number of samples is called the mixed strategy (MS). The strategy of migrating and fine-tuning all parameters related to the fault diagnosis model for the source domain using small samples is called the transfer strategy (TS). To evaluate the effectiveness of the model migration optimization strategy of fault diagnosis, the three small-sample processing strategies mentioned above are selected based on the multi-local feature fusion model. Then, the traditional strategy (MLMF-CS)-based, the hybrid strategy (MLMF-MS)-based, and the migration strategy (MLMF-TS)-based multi-local model bearing fault diagnosis algorithms were constructed. The hyperparameters of the model of the multi-local feature fusion for training in the above algorithm are set to be the same to guarantee a single variable, for following the principle of controlling variables.

**Figure 9:**
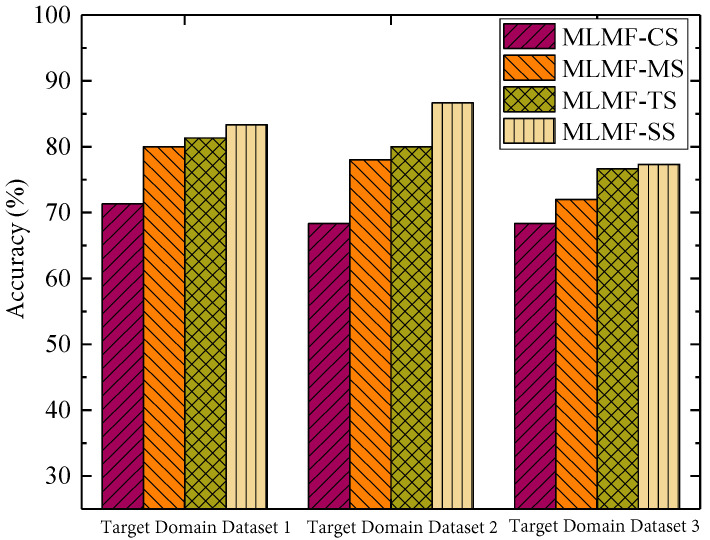
Algorithm fault diagnosis accuracy rate using different small-sample processing strategies.

Figure 9 shows the accuracy of the fault diagnosis using different small-sample processing strategies. It can be found that the proposed MLMF-SS algorithm has achieved good results in fault diagnosis experiments in different target fields, which shows that the migration optimization strategy in this algorithm is effective. That is, by using bearing datasets from different target domains for evaluation, it is demonstrated that the algorithm proposed in this paper can achieve high fault diagnosis accuracy regardless of the target domains with different deviation values, which verified the effectiveness of the maximum mean deviation algorithm in our proposed method. Among them, the MLMF-CS algorithm directly uses small sample data for the fault diagnosis. Hence, the model of fault diagnosis cannot effectively extract features. Ultimately, the accuracy of the fault diagnosis is much lower compared to other algorithms. The accuracy of the fault diagnosis of the MLMF-MS algorithm under different target domain datasets is significantly lower than that of the MLMF-SS algorithm and MLMF-TS algorithm. The MLMF-MS algorithm achieved 80% fault diagnosis accuracy only under the condition of target domain dataset 1. As the distribution difference between domains increases, the accuracy of the fault diagnosis algorithm shows a downward trend. This is because the algorithm directly trains the dataset in the source domain and that in the target domain together when the dataset of the target domain is scarce. This will inevitably cause the proportion of source domain data to be significantly higher than that of target domain data. Furthermore, it is obvious that the features extracted by the model of fault diagnosis are inclined to the source domain. Although the dataset of the source domain selected in this paper comes from similar bearings, the shallow features of the dataset for the bearing in the source and target domains have strong versatility. However, there are bound to be some gaps in its deep-seated features. Furthermore, as the difference in the distributions of the datasets in the source and the target domains increases, the difference in deep features extracted from the two datasets will also become more and more obvious. The mixed strategy clearly ignores this, which affects the extraction performance and diagnostic accuracy of the fault features. The MLMF-TS algorithm does not take into account the distribution differences between domains. In addition, it directly freezes and migrates all the network parameters and the network structure from the source to the target domain. Therefore, the fault diagnosis performance achieved by the algorithm still needs to be improved.

To further evaluate the performance of the migration optimization strategy, the MLMF-CS algorithm, that directly uses the bearing data of the target domain for fault diagnosis, is set as a benchmark for comparison. We compare our proposed MLMF-SS algorithm with the MLMF-TS algorithm, which achieved higher accuracy compared with MLMF-CS and MLMF-MS in the performance evaluation above. Due to the limited sample data of the test set, the diagnosis time for the algorithms is relatively short, which means they cannot effectively show the efficiency of the actual operation of the different algorithms. Therefore, this section evaluates the performance of each algorithm through the relative proportion of training time reduction, as shown in Table 3. Through comparison, it can be found that the two algorithms have improved the training efficiency. However, the training time reduction when using the MLMF-SS algorithm is about twice that of the MLMF-TS algorithm, which shows that our proposed algorithm has advantages in training efficiency.

### 5.3. Performance Evaluation of Fault Diagnosis Algorithms under Small Sample Conditions

To evaluate the MLMF-SS algorithm, the long short-term memory neural network (LSTM)-based bearing fault diagnosis method, the fault diagnosis algorithm for bearing using transfer component analysis deep belief network (TCA-DBN), and the deep transfer convolutional neural network (TCNN)-based algorithm are selected as the comparison algorithms. To confirm the stability and reliability in terms of the results obtained from the experiments, several repeated experiments are carried out independently for each algorithm, and the performance of the algorithm is judged according to the average result of the experiment.

**Figure 10:**
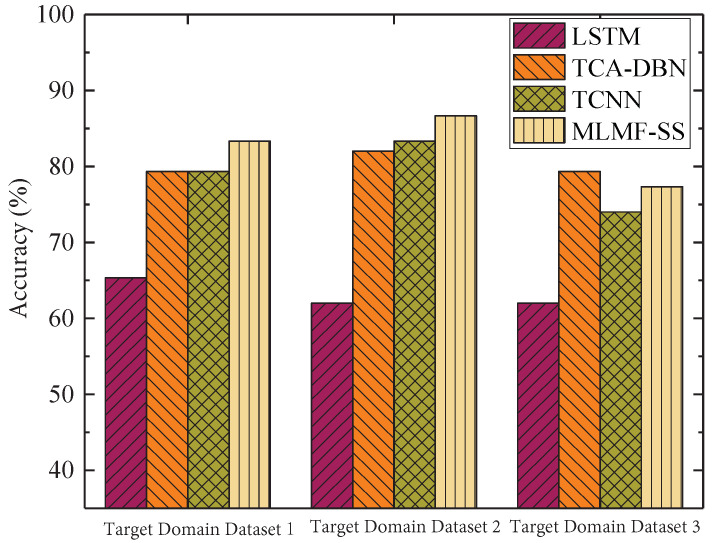
Accuracy of fault diagnosis for different algorithms.

Figure 10 shows the accuracy of the fault diagnosis for each algorithm. By comparing with the other three algorithms, it can be seen that the proposed algorithm has certain advantages in the accuracy index of fault diagnosis. It is slightly lower than the TCA-DBN algorithm only when selecting dataset 3 in the target domain. This shows that the MLMF-SS algorithm proposed in this paper makes full use of the data samples in the source domain and transfers the shallow features of the source domain to the target domain effectively. This guarantees the accuracy of the fault diagnosis using a small number of samples. The performance of the LSTM-based algorithm is unsatisfactory in its accuracy of the fault diagnosis; far inferior compared with the other three algorithms. This is because the algorithm directly trains the model without using migration learning or related migration optimization strategies. However, with a small number of samples, the number of training samples for the model is insufficient, which directly and seriously affects the ability of the model’s fault diagnosis and generalization algorithm performance. As mentioned above, the MMD values of the source domain and the target domain bearing datasets 1, 2, and 3 that we used were 0.110, 0.382, and 0.857, respectively. The difference between the source and target domain for dataset 3 is the highest. Since the TCA-DBN algorithm effectively transfers the feature samples in the source domain through the semi-supervised migration component analysis strategy, in turn, the degree of difference of the data in the source and target domains is reduced. Hence, the accuracy of fault diagnosis with scarce samples is improved to a certain extent for the TCA-DBN algorithm using dataset 3. Although the TCNN algorithm also performs feature migration, the algorithm does not comprehensively consider whether the degree of difference of the datasets in the source and target domains will affect the feature migration. Therefore, the algorithm shows a better diagnostic effect when the difference between the domains is small. However, when the difference between the domains is large, the ability of the algorithm to extract transfer features is insufficient. Therefore, the fault diagnosis is far inferior to that of the MLMF-SS algorithm and the TCA-DBN algorithm.

**Figure 11:**
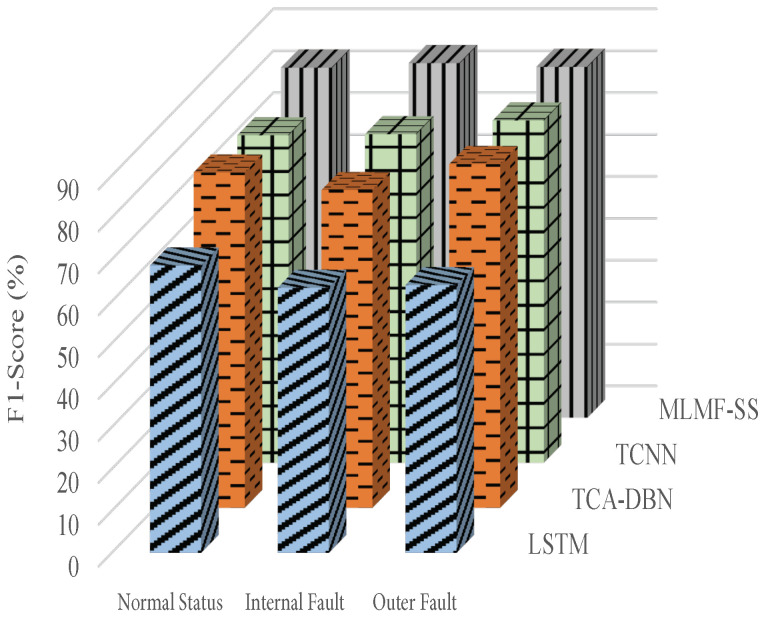
F1 score index when the target domain dataset 1 is selected.

**Figure 12:**
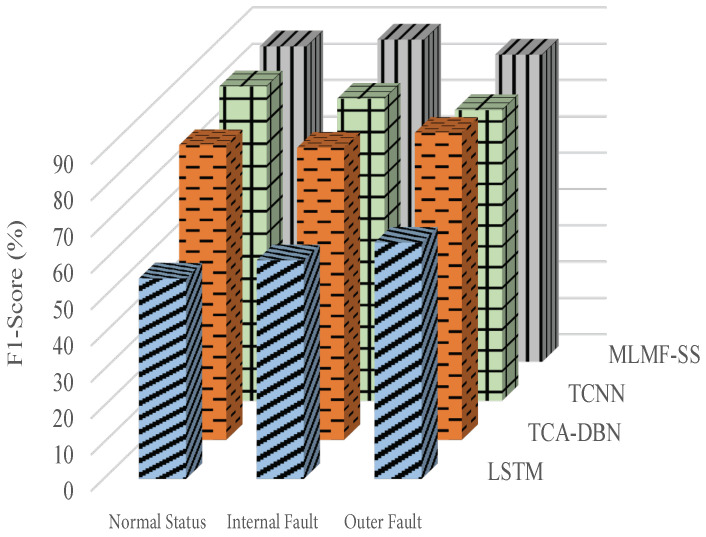
F1 score index when the target domain dataset 2 is selected.

**Figure 13:**
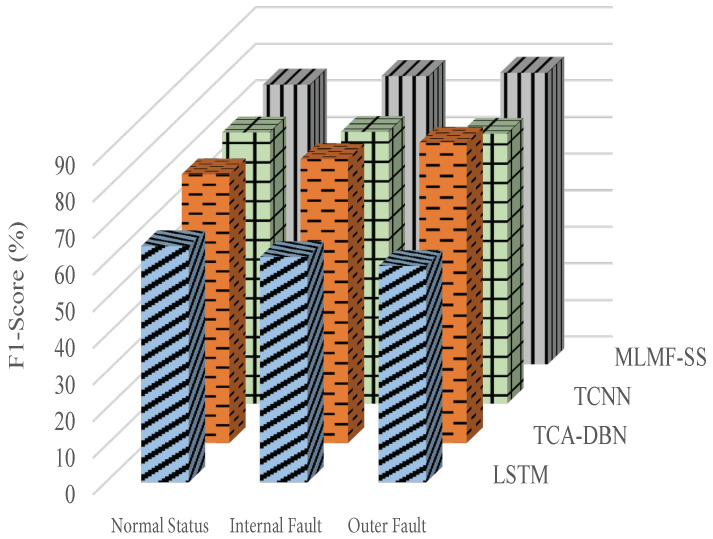
F1 score index when the target domain dataset 3 is selected.

Figure 11, Figure 12 and Figure 13 show the algorithms’ F1 score indicators when using the datasets 1, 2, and 3, respectively, of the target domain for experiments. In terms of F1 score index, the proposed MLMF-SS algorithm performs well, and the fluctuation range is relatively small under different bearing operating conditions. This shows that the comprehensive performance of the MLMF-SS algorithm is excellent. In addition, although the MLMF-SS algorithm has a slightly lower diagnostic accuracy than the TCA-DBN algorithm when experimenting with dataset 3 in the target domain, in terms of the F1 score under different operating states, the MLMF-SS algorithm is basically better than the TCA-DBN algorithm. Combining the F1 score index and the accuracy of the fault for each algorithm, a better performance is achieved for the MLMF-SS algorithm compared to other comparative algorithms.

## 6. Conclusions and Future Work

A small sample fusion-based multi-local model fault diagnosis algorithm (MLMF-SS) for bearings is proposed to solve the problem that the accuracy of fault diagnosis is low when the number of samples is small. MLMF-SS is based on a Bi-LSTM multi-local feature fusion model construction and migration optimization technology of a bearing small-sample fault diagnosis model based on the similarity migration measure. A performance evaluation showed the advantages of the proposed algorithm in both detection accuracy and the F1 score index. Specifically, firstly, the effectiveness of optimizing the migration of fault diagnosis models is evaluated. The experimental results show that the MLMF-SS method can improve the accuracy of diagnosis and reduce the model training time compared to MLMF-CS, MLMF-MS, and MLMF-TS. This indicates that our proposed algorithm has significant advantages in training efficiency. In addition, we evaluated and compared the accuracy of the proposed MLMF-SS with LSTM, TCA-DBN, and TCNN methods, as well as the F1 score using different datasets. The experimental results showed that the proposed method can improve the accuracy of diagnosis and the F1 score under any dataset.

The research work involved in this paper mainly focuses on fault diagnosis of single faults in industrial motor bearings. However, in actual industrial operations, the motor bearings are in complex environments such as variable load, variable speed, high temperature, and high pressure for a long time, and multiple faults may coexist. In this case, multiple source signals will interfere with each other, and the transmission path, speed fluctuations, and load changes in the signals will also have unpredictable effects on the bearing fault signal. As a result, the multi-source signals of the bearings will completely lose their original periodic variation pattern. In the future, relevant research will be carried out on the accurate separation of composite fault features of bearings, combining information fusion technology with composite fault technology. In addition, when dealing with the measurement problem of bearing data sample migration in this study, it is assumed that the contribution of each data sample to the intrinsic structural and distribution information of the dataset is the same. However, the contribution, sensitivity, and reliability of normal samples, single fault samples, and composite fault samples to the entire bureau are different. Therefore, how to reflect the differences in global metrics among different samples in the fault diagnosis process is also an important direction for future research.

## Figures and Tables

**Table 1:** The settings of the datasets for the source and target domains used in experiment.

Dataset Name	Rotating Speed (rmp)	Load (hp)	Damage Diameter (mm)	Operating Status	Sample Size
Dataset of source domain	1500	0	0.03	Normal status, internal fault, outer fault	250, 250, 250
Dataset 1 of target domain	1530	0	0.03	Normal status, internal fault, outer fault	100, 100, 100
Dataset 2 of target domain	1550	1	0.03	Normal status, internal fault, outer fault	100, 100, 100
Dataset 3 of target domain	1700	2	1	Normal status, internal fault, outer fault	100, 100, 100

**Table 2:** Network structure parameters of fault diagnosis model.

Network Layer Name	Activation Function	Number of Units
Bi-LSTM layer a	Tanh	32
Bi-LSTM layer b	Tanh	16
Attention mechanism layer	/	16
Fully connected layer	ReLU	32
Classification layer	Softmax	3

**Table 3:** The training time reduction using different strategies.

Name of Algorithm	MLMF-TS	MLMF-SS
Training time reduction ratio	15.3	29.0

## Data Availability

The data that support the findings of this study are available from the corresponding author upon reasonable request.
